# Short Read Alignment Based on Maximal Approximate Match Seeds

**DOI:** 10.3389/fmolb.2020.572934

**Published:** 2020-11-05

**Authors:** Wei Quan, Dengfeng Guan, Guangri Quan, Bo Liu, Yadong Wang

**Affiliations:** ^1^School of Computer Science and Technology, Harbin Institute of Technology, Harbin, China; ^2^Institute of Zoology, Chinese Academy of Sciences, Beijing, China

**Keywords:** whole-genome resequencing, next-generation sequencing, repeats, sequence alignment, FM-index

## Abstract

Sequence alignment is a critical step in many critical genomic studies, such as variant calling, quantitative transcriptome analysis (RNA-seq), and metagenomic sequence classification. However, the alignment performance is largely affected by repetitive sequences in the reference genome, which extensively exist in species from bacteria to mammals. Aligning repeating sequences might lead to tremendous candidate locations, bringing about a challenging computational burden. Thus, most alignment tools prefer to simply discard highly repetitive seeds, but this may cause the true alignment to be missed. Using maximal approximate matches (MAMs) as seeds is an option, but MEMs seeds may fail due to sequencing errors or genomic variations in MEMs seeds. Here, we propose a novel sequence alignment algorithm, named MAM, which can efficiently align short DNA sequences. MAM first builds a modified Burrows-Wheeler transform (BWT) structure of a reference genome to accelerate approximate seed matching. Then, MAM uses maximal approximate matches (MAMs) seeds to reduce the candidate locations. Finally, MAM applies an affine-gap-penalty dynamic programming to extend MAMs seeds. Experimental results on simulated and real sequencing datasets show that MAM achieves better performance in speed than other state-of-the-art alignment tools. The source code is available at https://github.com/weiquan/mam.

## 1. Introduction

The development of next-generation sequencing (NGS) technologies has led to a rapid decline in the sequencing cost and had a tremendous impact on genomic research (Morozova and Marra, 2008; Reinert et al., 2015). There has been an intense effort in recent years to develop computational methods and applications to meet the increasing demands for sequencing data analysis (Flicek and Birney, 2009). One of these fundamental tasks is sequence alignment. Sequence alignment is one of the most critical steps necessary to process NGS data, and the alignment accuracy has a very large impact on downstream applications, such as variant calling (Dalca and Brudno, 2010), quantitative measurement of RNA-seq (Pepke et al., 2009), eQTL analysis (Wang et al., 2019), and metagenomic analysis (Breitwieser et al., 2019; Cheng et al., 2020). During the past decades, many alignment methods have been proposed to improve the efficiency and accuracy of sequence alignment, including but not limited to Maq (Li et al., 2008a), SOAP (Li et al., 2008b), Bowtie (Langmead et al., 2009), BWA (Li and Durbin, 2009), and mrsFAST (Hach et al., 2010). With these developments, the performance of alignment tools has been greatly improved with respect to speed, sensitivity and accuracy (Li et al., 2009; Schadt et al., 2010; Langmead and Salzberg, 2012; Xiao et al., 2017). However, aligning repetitive DNA sequences accurately to the reference genome remains a major issue.

Repetitive DNA sequences are multiple copies of sequences with high similarity that occur throughout the genome. Repetitive DNA sequences are highly abundant in a broad range of species, from bacteria to mammals. For example, nearly 40% of bacterial genomes and 50% of human genomes are composed of repetitive sequences (Treangen and Salzberg, 2012). These repetitive patterns could cause several computational challenges for sequence alignment, which would result in loss of information related to essential biological phenomena. From a computational perspective, repeats may bring out ambiguous candidate positions, which will decrease alignment speed. In recent years, several alignment tools use maximal exact matches (MEMs) or super maximal exact matches (SMEMs) to reduce the number of candidate positions (Liu and Schmidt, 2012; Li, 2013). However, these approaches may reduce the sensitivity in complex genomic regions.

In this article, we build a novel index, named MAM-index, for a reference genome based on a modified Burrows-Wheeler transform (BWT) structure. MAM-index combines the conventional FM-index and an auxiliary data structure, which accelerates approximate string matching. We propose a MAMs based seeding algorithm to calculate the candidate locations. We present a novel sequence alignment tool, named MAM, that can efficiently align highly repetitive DNA sequences by using maximal approximate matches (MAMs) as seeds. To evaluate the performance of MAM, we performed alignment experiments on both simulated and real datasets, and compared our results with those of Bowtie and BWA-MEM. Experiments on simulated and real sequencing datasets show that MAM achieves better performance in speed than the other alignment programs.

## 2. Materials and Methods

### 2.1. Overview of MAM

**Figure 1:**
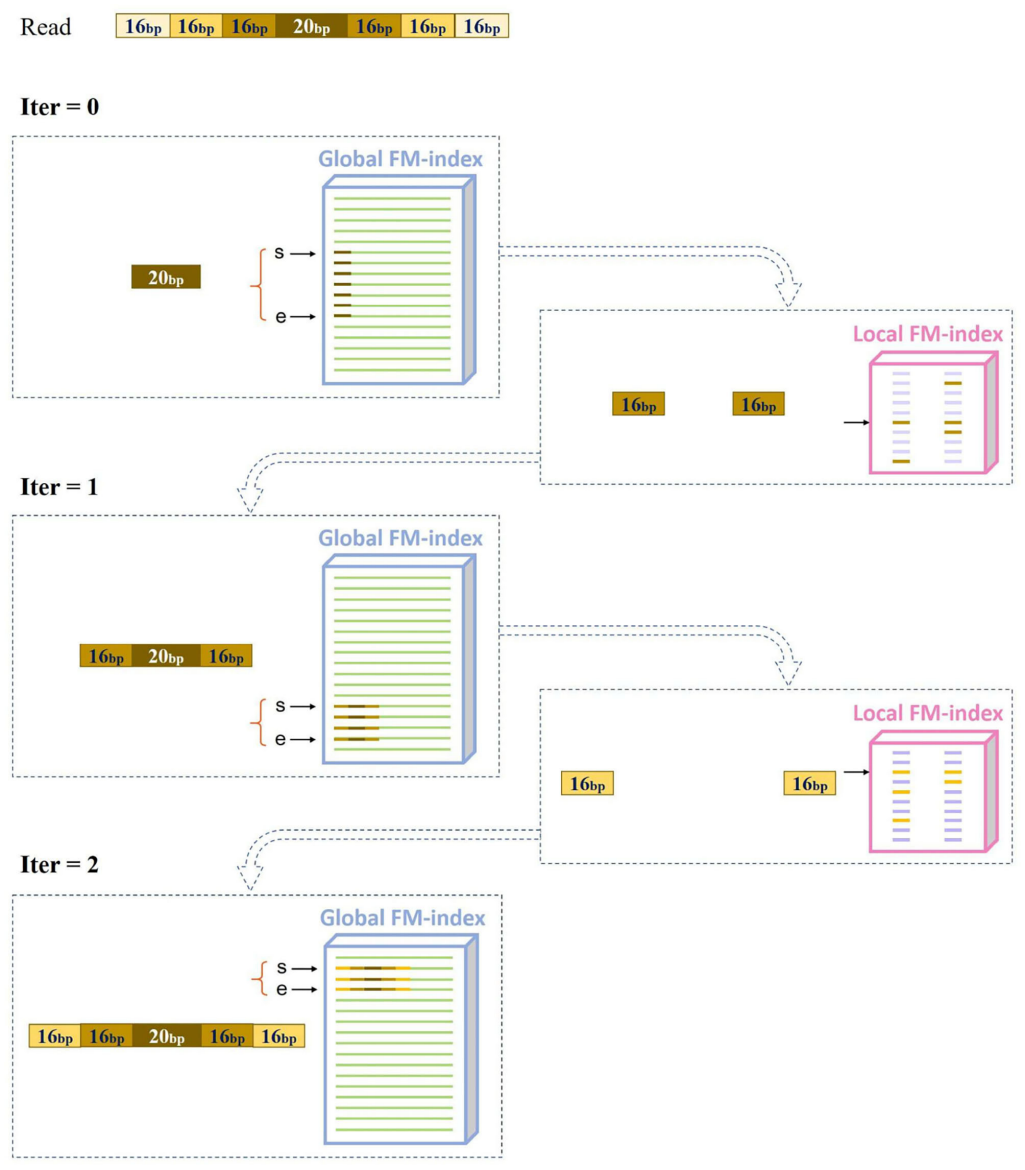
Overview of maximal approximate match seeding.

MAM uses a hierarchical index (MAM-index), which consists of two types of indexes: a global index (global FM-index) and numerous local indexes (local FM-indexes). MAM follows the canonical seed-and-extend paradigm. In the seeding stage, it initially seeds an alignment with exact matches of length *l*_*init*_ via the global FM-index. To reduce ambiguous candidate locations caused by highly repetitive seeds, we introduce maximal approximate matches as seeds to re-seed all initial seeds with more than *m* occurrences. The overview of MAM seeding is shown in Figure 1: (1) Search a *l*_*init*_ = 20 bp initial seed based on the global FM-index, and obtain global suffix array interval [*bg, ed*). (2) Find the local FM-index that corresponds to suffix array interval [*bg, ed*). (3) Use local FM-index to obtain the *l*_*ext*_ = 16 bp extended seed. (4) Repeat 3 until the seed is not extensible. After the seeding stage, we use affine-gap-penalty dynamic programming (DP) to extend all MAMs seeds with no more than *m* occurrences, and perform the best alignment.

### 2.2. Construction of the MAM-Index

The structure of the MAM-index consists of two parts: a global index (global FM-index) and numerous local indexes (local FM-indexes). A conventional FM-index is employed as the global index part of the MAM-index. The FM-index was proposed by Paolo Ferragina and Giovanni Manzini in 2000 (Ferragina and Manzini, 2000). The FM-index is a self-indexing index based on the Burrows-Wheeler transform (BWT), and it can be used to efficiently find the occurrences of a pattern in the case of low RAM. Below, we will focus on the construction of the local index.

The local index consists of a BWT-like data structure (sBWT) and a relation array (RA). The sBWT is a variation of the BWT, which is used to index multiple strings of equal length. Given a seed, we employ an sBWT to index predecessor sequences and successor sequences of the seed and a RA to store the relation information between successor sequences and predecessor sequences of the seed.

#### 2.2.1. Construction of the sBWT

##### 2.2.1.1. Building predecessor and successor sets

Given the i-th iteration seed *s*_*i*_, which occurs more than *k* times in the reference genome sequence, the *l*_*ext*_ length predecessor sequences of *s*_*i*_ are the *l*_*ext*_ length predecessors of *seed*_*i*_ at all occurrences of *s*_*i*_, denoted *pred*(*s*_*i*_, *l*_*ext*_). Similarly, the *l*_*ext*_ length successor sequences of *s*_*i*_ are the *l*_*ext*_ length successors of *s*_*i*_ at all occurrences of *s*_*i*_, denoted *succ*(*s*_*i*_, *l*_*ext*_).

**Figure 2:**
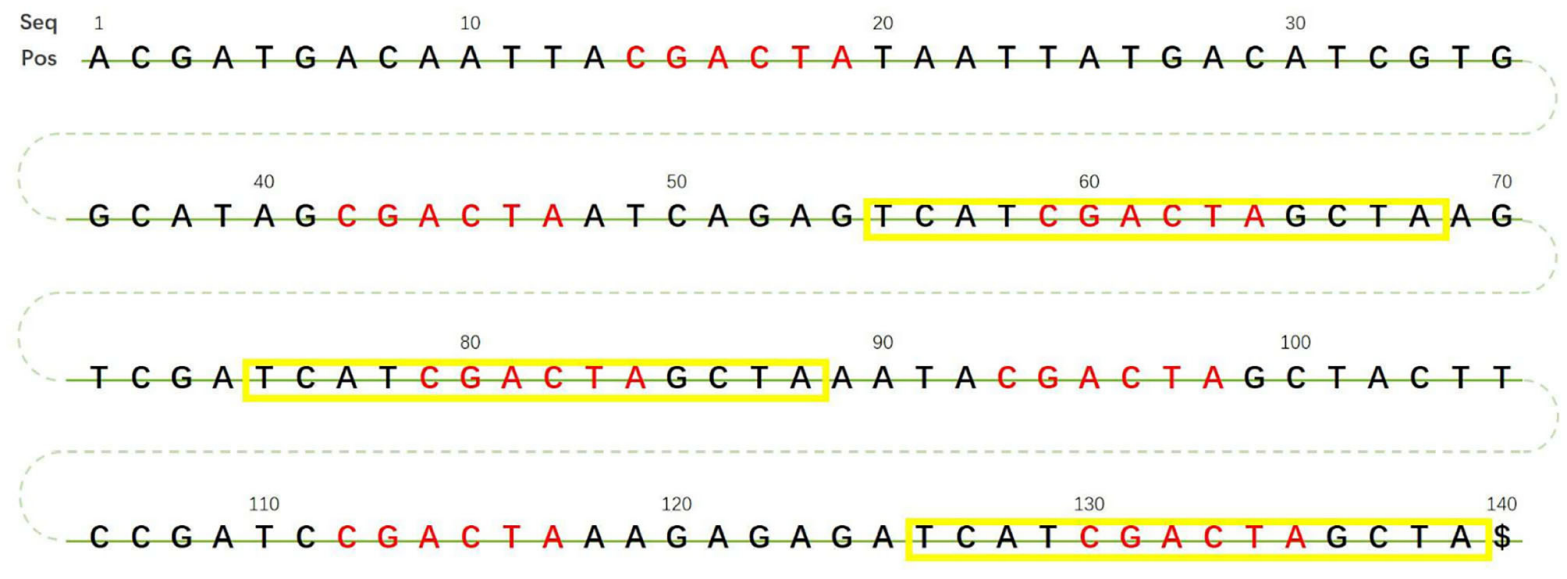
Example 140 bp length reference sequence.

The predecessor sequence set construction algorithm is shown in **Algorithm 1**. Similarly, the successor sequence set construction algorithm can be implemented by modifying the code for fetching local sequences from the reference genome sequence. To better illustrate the algorithm, we use a 140 bp reference genome sequence as an example; see Figure 2.

**Algorithm 1:**
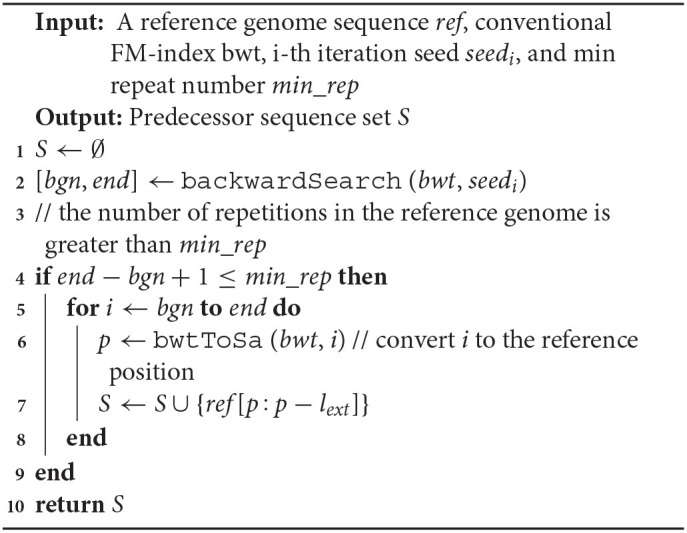
genPred

**Figure 3:**
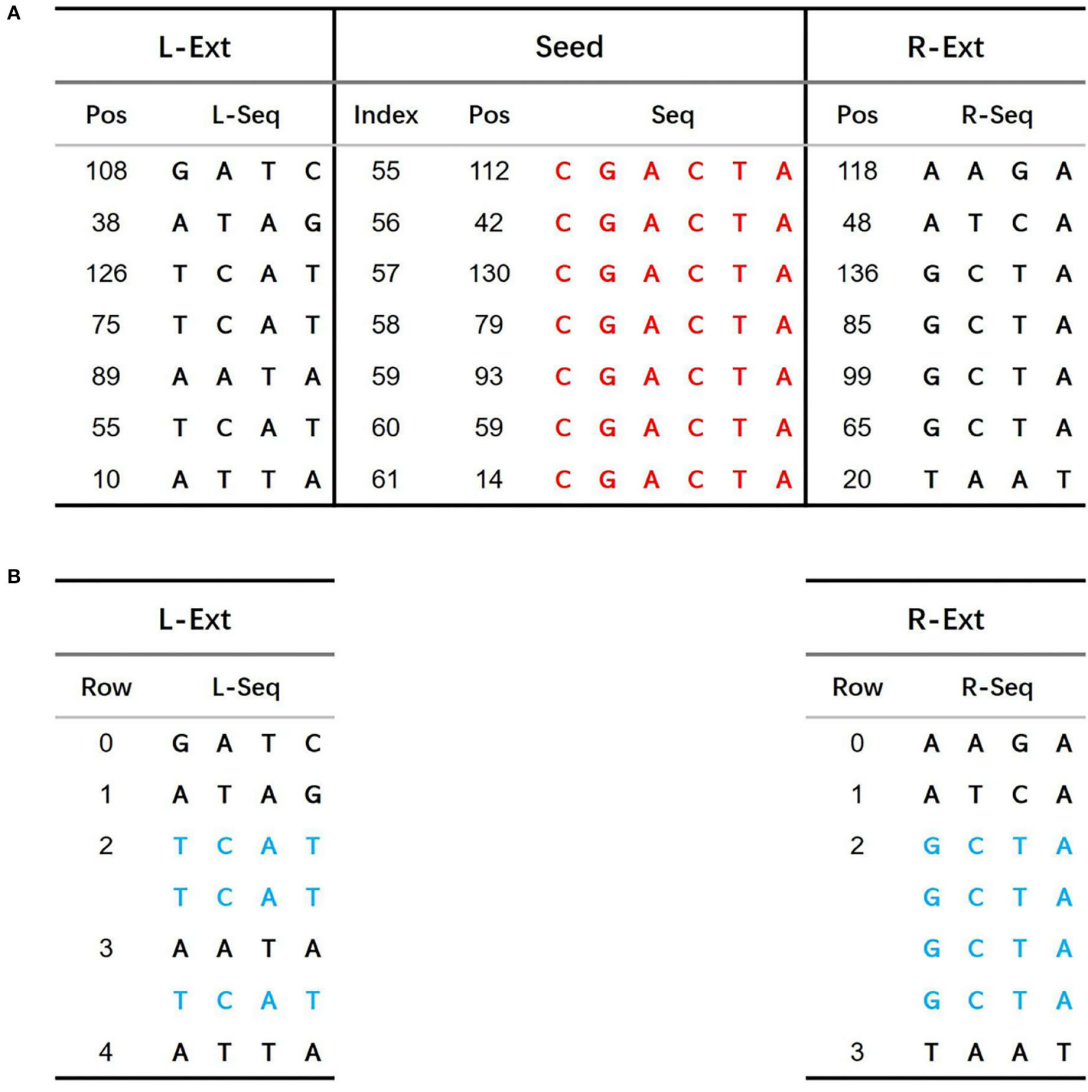
**(A)** Predecessor sequences and successor sequences of CGACTA. **(B)** Filter duplicated sequences in predecessor sequences and successor sequences.

In Figure 2, sequence *CGACTA* marked in red is an initial iteration seed with 7 occurrences in the 140 bp reference genome. The predecessor sequences and successor sequences of *CGACTA* are shown in Figure 3A. The L-Ext column lists the predecessor sequences of *CGACTA*, and the R-Ext column lists the successor sequences of *CGACTA*. The Pos sub-column lists the position of the sequence in the reference genome sequence. For duplicate sequences, only one copy of the duplicate sequences is stored in the predecessor sequence set; see Figure 3B. In Figure 3B, the local sequence *TCAT* in L-Ext.

##### 2.2.1.2. Building sBWT for predecessor and successor sets

**Figure 4:**
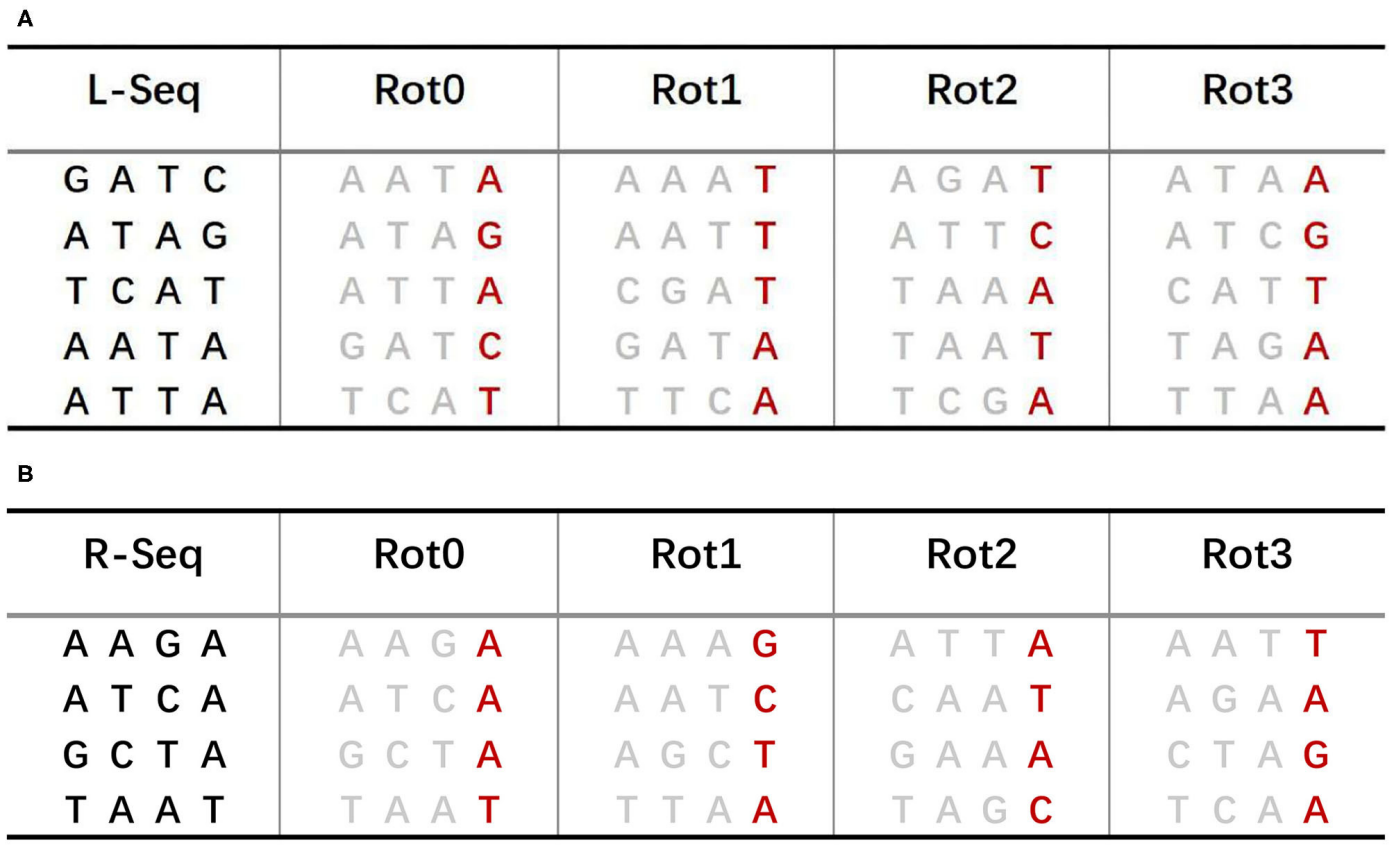
**(A)** sBWT of predecessor sequences. **(B)** sBWT of successor sequences.

The sBWT of predecessor and successor sequences is shown in Figures 4A,B, respectively. The construction process of sBWT is similar to that of the Burrows-Wheeler matrix. The *i*-th iteration transform is performed by sorting local sequences of *i* rotations in lexicographic order. The L-Seq column in Figure 4A is the predecessor sequences set, and Rot0 is the 0-th iteration transform of L-Seq. The construction algorithm of the sBWT is shown in **Algorithm 2**.

**Algorithm 2:**
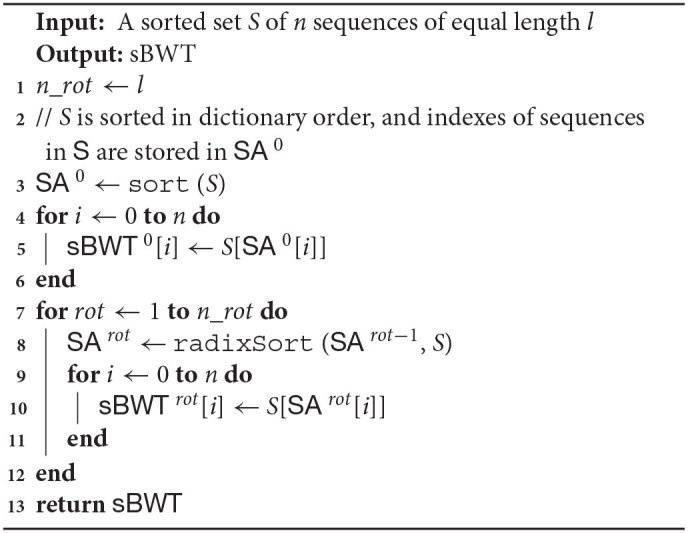
buildSbwt

#### 2.2.2. Construction of the Relation Array

sBWT can be used to find the occurrences of predecessor and successor sequences. However, the searched predecessor and successor sequences may not be proper paired, which may lead to ambiguous seed extension. For this reason, we use a relation data structure (RA) to store relation information between the predecessor and successor sequences.

**Figure 5:**
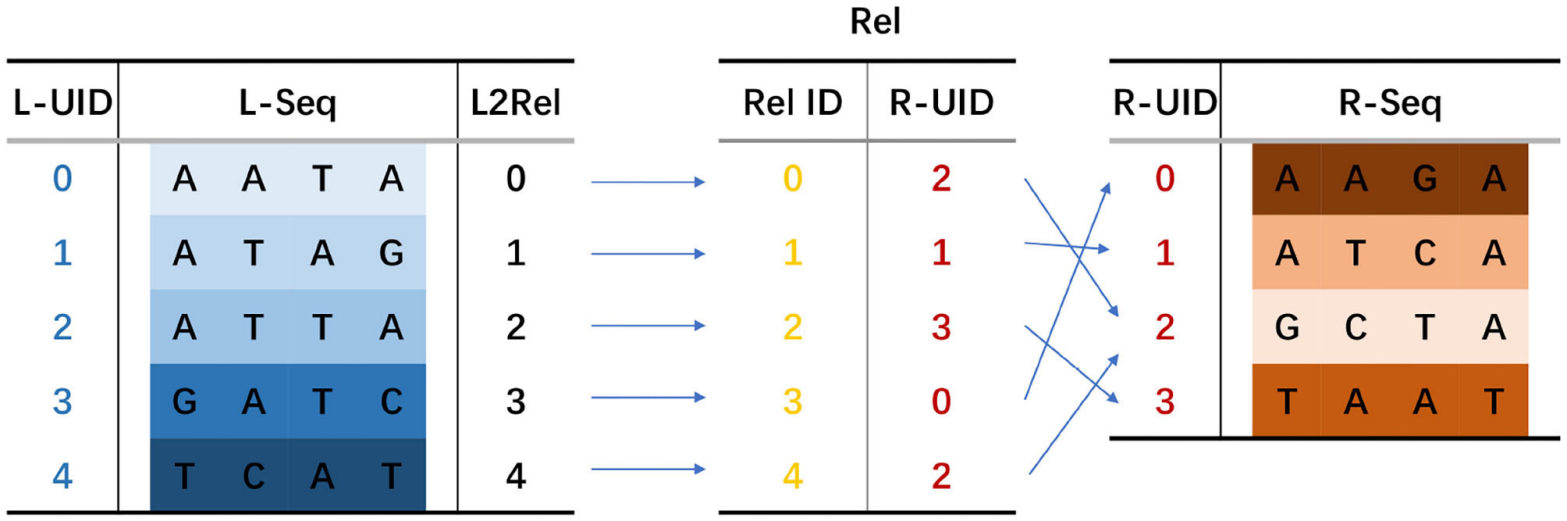
Relation array of seed *CGACTA*.

The RA consists of *LtoRel* and *Rel*. *LtoRel*[*i*] stores the initial index of the predecessor sequence in *Rel*. Given an i-th iteration seed *s*_*i*_ and a next iteration seed *s*_*i*+1_ = *pred*(*s*_*i*_) + *s*_*i*_ + *succ*(*s*_*i*_), if *s*_*i*+1_ occurs in the reference genome sequence, then *pred*(*s*_*i*_) and *succ*(*s*_*i*_) can be searched via sBWT. Supposing that *pred*(*s*_*i*_) is *rank*_*pred*_ ranked in all predecessor sequences and *succ*(*s*_*i*_) is *rank*_*succ*_ ranked in all successor sequences, *rank*_*succ*_ should be a value of *LtoRel*[*j*] for *j* in the range [*rank*_*pred*_, *rank*_*pred*_ + 1). *Rel*[*i*] stores the pointer to *s*_*i*+1_ in the MAM-index. With *Rel*[*i*], the seed jumps to the next iteration. The RA of seed *CGACTA* is shown in Figure 5.

### 2.3. Alignment With MAM

MAM follows the canonical seed-and-extend paradigm. MAM generates every *x* nt exact match seeds in reads. If seeds occur more than *k* times, MAM iteratively extends seeds by finding approximate matches in both directions until the occurrences of seeds are less than *k* or seeds are not extensible. After seeding, MAM generates locations of seeds. All locations are sorted in ascending order, and all locations whose distances are less than *d* are placed within the same chain. Then, all chains are sorted by seeds number in descending order. After chains are sorted, MAM performs a striped Smith-Waterman algorithm for the top *k* chains. We choose the best score location as the best alignment.

The algorithm of alignment with MAM is shown in **Algorithm 3**. In **Algorithm 3**, the function backwardSearchGlobal is a conventional backward search algorithm, and the function backwardSearchLocal is shown in **Algorithm 4**. C (*sbwt*^*i*^, c) is a function that contains the number of occurrences of lexically smaller characters in the i-th rotation sBWT. The function Occ (*sbwt*^*i*^, k, c) is the number of occurrences of character c in the *sbwt*^*i*^[1 : *k*].

**Algorithm 3:**
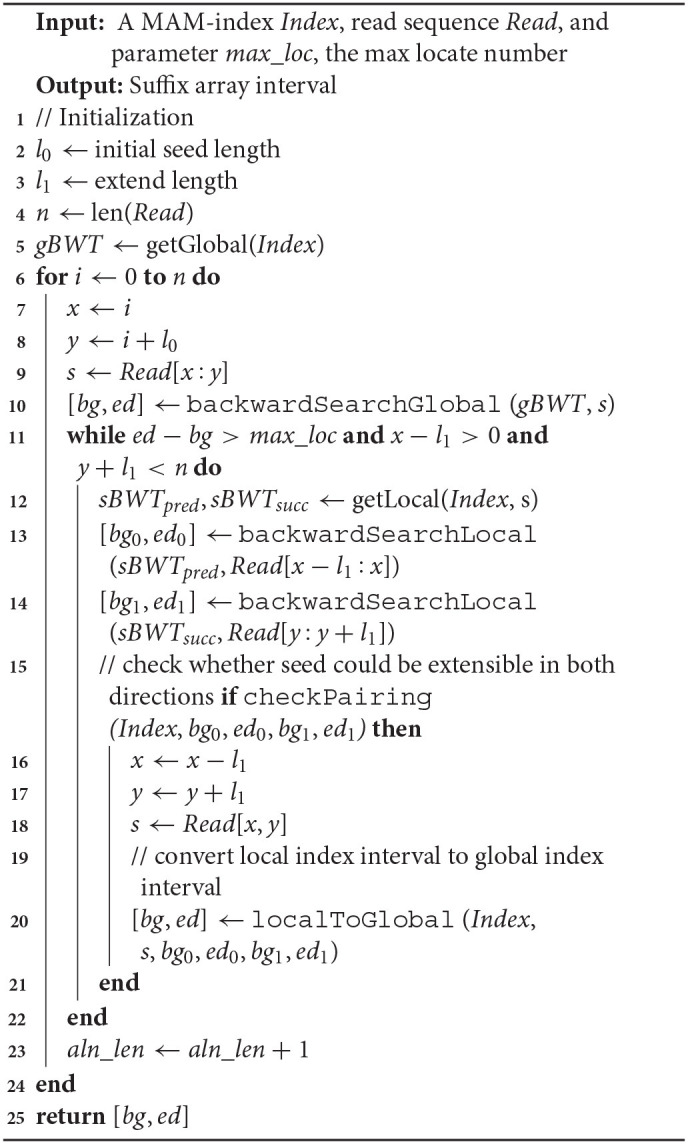
Alignment with MAM

**Algorithm 4:**
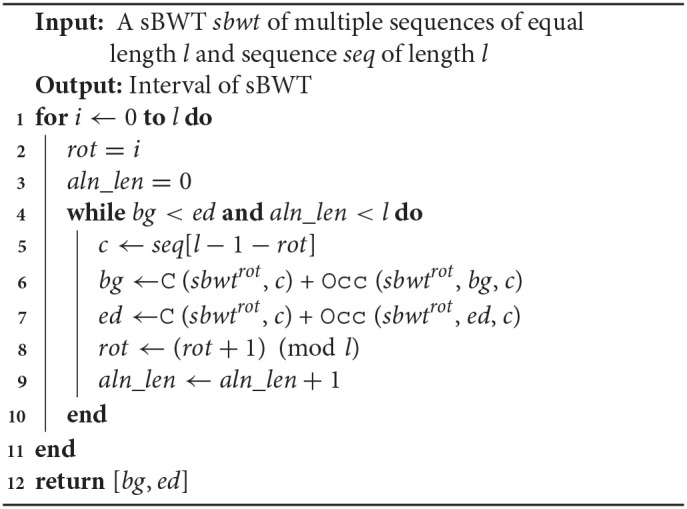
backwardSearchLocal

## 3. Results

We have implemented MAM to align short reads to a reference genome. The default output format is SAM format. MAM is distributed under the GNU General Public License (GPL). The source code is available at https://github.com/weiquan/mam.

The performance of MAM has been compared with those of the he most widely used alignment alignment tools BWA-MEM (version 0.7.17) and BOWTIE2 (version 2.3.5). All aligners were tested on two simulated datasets and two high-throughput sequencing (HTS) datasets to assess their speed, sensitivity, and accuracy. All benchmarks were conducted on a desktop computer with 32 GB of RAM and a 3.30 GHz Intel i9-7900X processor with a total of 10 CPU cores running Linux Ubuntu 18.04.

### 3.1. Evaluation on Simulated Datasets

We simulated 4 million 100 and 150 bp Illumina-like reads from the human genome GRCh38 using Mason2 (Holtgrewe, 2010) with a 0.1% SNP mutation rate, a 0.02% indel mutation rate and a 2% sequencing base error rate. We ran MAM, BWA-MEM and BOWTIE2 with the default settings. A read is defined as a mapped read if the read is mapped with at least one alignment. An alignment is defined as a good alignment if the alignment position is the true position. The sensitivity and accuracy are defined as the following percentages:

sensitivity=#mapped/#reads×100%

accuracy=#good/#mapped×100%

Table 1 shows that BWA-MEM (100.00%) is more sensitive than MAM (99.90–99.97%) and BOWTIE2 (99.01–99.39%) on both the sim-100 and sim-150 dataset. BWA-MEM(95.06–96.24%) is the most accurate on both the sim-100 dataset and the sim-150 dataset. With respect to speed, MAM is the fastest on both the sim-100 dataset and sim-150 dataset. On memory, BOWTIE2 uses (3.22–3.23 GB), smaller than BWA-MEM (5.26–5.39 GB), and MAM (16.54–16.59 GB).

**Table 1:** Evaluation on simulated data.

**Program**	**sim-100**				**sim-150**			
	**Sen (%)**	**Acc (%)**	**Time**	**Mem**	**Sen (%)**	**Acc (%)**	**Time**	**Mem**
MAM	99.90	94.65	12m28s	16.54GB	99.97	95.96	23m13s	16.59GB
BWA-MEM	100.00	95.06	16m20s	5.39GB	100.00	96.24	39m43s	5.26GB
BOWTIE2	99.01	93.88	22m56s	3.22GB	99.39	95.28	25m46s	3.23GB

*Sen, alignment sensitivity; Acc, alignment accuracy; Mem, the peak memory usage of the tool*.

### 3.2. Evaluation on HTS Datasets

We benchmarked all aligners on two HTS datasets to assess the performance on real datasets. We mapped 4 million 100 bp reads sequenced with the Illumina HiSeq 2000 (SRA ID: ERR037900) and 4 million 148 bp reads sequenced with the Illumina HiSeq 2000 (SRA ID: SRR1766443) to the human reference genome (GRCh38). All aligners were run with the default settings. As the true alignment locations of reads are not known in real datasets, we define that an alignment is a good alignment if the alignment score between the read and the reference is higher than 85% of the highest score (e.g., if the alignment score of a 100 bp read is higher than 85, it is regarded as a perfect alignment).

The results are shown in Table 2. Table 2 shows that MAM (95.89%) is the most accurate on the real-100 datasets, and BOWTIE2 (97.32%) is the most accurate on the real-148 datasets. BWA-MEM (99.51–99.99%) is the most sensitive on both the real-100 and real-148 datasets. With respect to speed, MAM (8m59s) is the fastest on the real-100bp dataset, and BWA-MEM (21m57s) is the fastest on the real-148 dataset. On memory, BOWTIE2 uses (3.22–3.23 GB), smaller than BWA-MEM (5.24–5.39 GB), and MAM (16.52–16.59 GB).

**Table 2:** Evaluation on real data.

**Program**	**real-100**				**real-148**			
	**Sen (%)**	**Acc (%)**	**Time**	**Mem**	**Sen (%)**	**Acc (%)**	**Time**	**Mem**
MAM	99.83	95.89	8m59s	16.52GB	98.85	96.21	24m37s	16.59GB
BWA-MEM	99.99	95.87	18m18s	5.39GB	99.51	96.27	21m57s	5.24GB
BOWTIE2	99.62	94.54	13m37s	3.22GB	98.11	97.32	24m29s	3.23GB

*Sen, alignment sensitivity; Acc, alignment accuracy; Mem, the peak memory usage of the tool*.

## 4. Discussion

Enormous amounts of short read aligners have been developed for fast and accurate alignment of reads to a reference genome. However, aligning repetitive DNA sequences to the reference genome is still a concern. Some of the aligners employ MEMs seeds to reduce candidate locations in repetitive regions. However, MEMs seeds may fail due to genomic variations and sequencing errors. To this purpose, we use MAMs seeds to filter candidate locations in the seeding stage. Although MAMs seeds could be searched via the conventional FM-index, it is ineffective.

Herein, we propose a variation FM-index (MAM-index) to search MAMs seeds quickly and present a short read alignment tool (MAM). We have demonstrated the performance of MAM on aligning sequences to the human genome, and compared it with the most widely used alignment tools, BWA-MEM and BOWTIE2. The result shows MAM is more efficient than BWA-MEM and BOWTIE2 with similar accuracy and sensitivity. In addition, accuracy and sensitivity of MAM could be improved by using shorter initial seed length, which means MAM has the potential to align sequences to complex genomic regions. Although MAM requires more memory than BWA-MEM and BOWTIE2, memory is not a practical concern on modern computer servers.

## Data Availability Statement

All datasets generated for this study are included in the article/supplementary material.

## Author Contributions

WQ and GQ designed the algorithm of indexing and seeding. WQ implemented the algorithm and performed in silico experiments. DG co-wrote the manuscript and co-performed the experiments. BL and YW revised the manuscript and provided funding support.

## Conflict of Interest

The authors declare that the research was conducted in the absence of any commercial or financial relationships that could be construed as a potential conflict of interest.
